# Defragmentation in ablative AF treatment: is our knowledge too fragmented?

**DOI:** 10.1007/s12471-013-0494-8

**Published:** 2013-11-21

**Authors:** N. M. van Hemel, J. M. T. de Bakker

**Affiliations:** 1 Department of Cardiology, University Medical Center Utrecht, Heidelberglaan 100, 3584 CX Utrecht, the Netherlands; 2 Department of Physiology, University Medical Center Utrecht, Heidelberglaan 100, 3584 CX Utrecht, the Netherlands

Our current knowledge of the arrhythmic genesis of atrial fibrillation (AF) converges to the AF multiple wavelets concept of Moe and Allessie of the 1970s, which was further elaborated by many other investigators. Around 1990, spontaneous automaticity arising in muscular sleeves of pulmonary veins was documented by Haissaguerre and his rhythm experts of Bordeaux. Therefore, it made sense that after failed antiarrhythmic drug treatment to prevent or suppress AF, these concepts were translated into invasive therapy. Around 1985, cardiac surgeons Cox and Guiraudon were the first to eliminate the type of AF based on the multiple wavelets concept with different cut-and-sew approaches. Due to the large impact of surgery, catheter ablation to eliminate automaticity with pulmonary vein isolation and (gradually more) atrial ablation lines followed. The assumption that the percutaneous approach would be a promising, less aggressive alternative for surgery, however, evaporated due to the inferior long-term results compared with current minimal invasive surgery [1]. These disappointing results conceivably prompted many groups to explore additional measures for the eradication of AF, such as energy sources different from radiofrequency, ablation of the cardiac nervous ganglia and elimination of zones of slow or non-uniform conduction guided by voltage differences or fragmentation mapping of atrial tissue [2].

Fragmented atrial signals and low electrogram voltages are often recordable in AF [3] for example after repair of congenital heart disease and various AF types and manifestations [2]. Their documentation with modern automated signal analysis and 3D mapping raises questions such as their relation to the amount and depth of scarred tissue, stability during sinus rhythm (P wave characteristics) and/or other types of AF and/or atrial tachycardia. Most crucial for therapeutic application is their reproducible contribution to the onset and perpetuation of AF with critical conduction pathways. Studies showed that the incidence and duration of fragmentation are larger in ‘chronic’ compared with ‘paroxysmal’ AF patients and are also related to ageing, left atrial dilatation, shorter excitable periods and non-uniform conduction or anisotropy in atrial tissue. Non-uniform conduction is caused by fibrosis and observed at higher ages and increased atrial pressure. Evidence also exists that mapping of fragmented P waves in sinus rhythm also points to patients prone to or known to have atrial tachyarrhythmias, reflecting functional or structural changes. These observations strongly suggest that fragmentation can potentially guide the ablation procedure in drug refractory AF.

In this issue, De Vries et al. [4]. report on a comparative study of RF catheter ablation with pulmonary vein isolation and left atrial lines with or without additional ablative ‘shaving’ of atrial sites with fragmented signals in patients with chronic AF, the so-called defragmentation method. Because this approach takes more time and burning, it is not surprising that more (minor) complications occurred in the defragmentation group. However, it is unclear why defragmentation could not bring out a better result in terms of suppression of AF at 3, 6 and 12 months after the procedure. The report by De Vries et al. [4]. cannot be compared with the results of previous studies, because the applied method of additional defragmentation has not been carried out before. The authors attributed the lack of increased success to resumed conduction from the automatic foci in the pulmonary veins or through the atrial ablation lines, despite all procedural measurements of (successful) conduction block. Furthermore they discussed the risk of ‘insufficient defragmentation’, an ill-defined term, and speculated whether more defragmentation would have ameliorated the outcome. Unfortunately, the authors did not correlate the findings of atrial mapping with the number of recurrences of AF.

Finally, weighting the 12-month AF outcome versus complication rate and prolonged procedural time, they concluded that defragmentation should not be considered a routine part of catheter ablation for AF.

It should be emphasised that this advice results from a retrospective study of two Dutch invasive arrhythmia groups that undermines the power of this advice. Second, because defragmentation is guided by analysis of endocardial atrial signals, information about the epicardial conduction is missing. Simultaneous endocardial and epicardial recordings of atrial arrhythmias in the dog heart showed a clear discordant activation. Another study in the goat showed that this dissociation is related to an increase in separation between the epicardial layer and endocardial bundles and it is suggested that the thin epicardial layer of the atrial wall can conduct fibrillatory waves [5]. This layer is probably not (always) reachable or too risky with current ablative techniques. Third, our knowledge of the degree of structural and functional temporo/spatial differences of (slow) conduction or block and its relation to the ‘severity’ of AF is limited. More experimental work is necessary to understand the significance of fragmented signals in the individual patient at a certain time of the AF disease.

Therefore, before classifying defragmentation a bridge too far, more investigations are needed. We learn little from success but more from failures, reason to thank the authors and the Journal for publication of this initially unsuccessful therapeutic method. The investigators are encouraged to contact the experimental lab to study fragmented atrial signals and to consider a prospective, well-defined, study in a comparable group of AF patients after they have gained more insight into the role of these fragmented atrial signals.
